# Supplementary material for: Optimized Selection of Water Resource Allocation Schemes Based on Improved Connection Entropy in Beijing’s Southern Plain
Source: Entropy (Basel). 2022 Jul 1;24(7):920. doi: 10.3390/e24070920 (PMC9319829; doi:10.3390/e24070920)
Supplement: Supplementary file 1 [file entropy-24-00920-s001.zip › entropy-1766631-supplementary.pdf]

Table S1 Connection number of indicators of the actual water-use schemes from 2006 to 2012

Evaluation indicators	2006	2007	2008	2009	2010	2011	2012
X1	0+0.7681I+0.2319J	0+0.7752I+0.2248J	0+0.7617I+0.2383J	0.1913+0.8087I+0J	0.4325+0.5675I+0J	0.3422+0.2976I+0.3602J	0.3102+0.2948I+0.3950J
X2	0+0.5674I+0.4326J	0+0.5704I+0.4296J	0+0.5569I+0.4431J	0+0.5157I+0.4843J	0+0.5002I+0.4998J	0.9365+0.0314I+0.031J	0.8504+0.0464I+0.1032J
X3	0+0.6411I+0.3589J	0.8436+0.0753I+0.0811J	0+0.5017I+0.4983J	0+0.6299I+0.3701J	0+0.6028I+0.3972J	0+0.5555I+0.4445J	0.7793+0.092I+0.1285J
X4	0+0.5498I+0.4502J	0.2522+0.2517I+0.4961J	0.4707+0.5293I+0J	0+0.6693I+0.3307J	0+0.5055I+0.4945J	0.3360+0.2859I+0.3781J	0.3512+0.2921I+0.3567J
X5	0+0.8239I+0.1761J	0+0.5025I+0.4975J	0.3258+0.3125I+0.3617J	0+0.8096I+0.1904J	0+0.6518I+0.3482J	0.3507+0.3209I+0.3284J	0.3092+0.3068I+0.3840J
X6	0+0.7940I+0.2060J	0+0.6317I+0.3683J	0+0.6152I+0.3848J	0+0.6040I+0.3960J	0+0.5039I+0.4961J	0.3740+0.3051I+0.3209J	0.3742+0.3051I+0.3207J
X7	0+0.7815I+0.2185J	0+0.5451I+0.4549J	0+0.5027I+0.4973J	0+0.6636I+0.3364J	0+0.5223I+0.4777J	0.3564+0.3082I+0.3354J	0.3538+0.3076I+0.3386J
X8	0+0.8239I+0.1761J	0+0.5023I+0.4977J	0.3257+0.3124I+0.3619J	0+0.8095I+0.1905J	0+0.6517I+0.3483J	0.3506+0.3209I+0.3285J	0.3091+0.3068I+0.3841J
X9	0+0.5796I+0.4204J	0+0.5905I+0.4095J	0.2513+0.2477I+0.500J	0+0.6624I+0.3376J	0+0.6633I+0.3367J	0.3173+0.2401I+0.4426J	0.4488+0.5512I+0J

Table S2 Connection number of indicators of the actual water-use schemes from 2013 to 2016 and the optimal water resources allocation schemes for the three groundwater exploitation modes

Evaluation indicators	2013	2014	2015	2016	Groundwater extraction mode 1	Groundwater extraction mode 2	Groundwater extraction mode 3
X1	0.4632+0.5368I+0J	0.2943+0.2935I+0.4122J	0.3919+0.6081I+0J	0.3957+0.3021I+0.3021J	0.3957+0.3021I+0.3022J	0.3957+0.3021I+0.3022J	0.3958+0.3021I+0.3021J
X2	0.8496+0.0465I+0.1039J	0.6029+0.0893I+0.3078J	0.4996+0.5004I+0J	0.4915+0.5085I+0J	0.1658+0.1652I+0.6690J	0.4953+0.5047I+0J	0.4918+0.5082I+0J
X3	0.7364+0.1032I+0.1604J	0.7040+0.1117I+0.1843J	0.2438+0.2315I+0.5247J	0.4780+0.5220I+0J	0.4780+0.5220I+0J	0.4780+0.5220I+0J	0.4780+0.5220I+0J
X4	0+0.6603I+0.3397J	0+0.5136I+0.4864J	0.3554+0.2938I+0.3508J	0.2822+0.2640I+0.4538J	0.1853+0.8147I+0J	0.1854+0.8146I+0J	0.1718+0.8282I+0J
X5	0.4129+0.5871I+0J	0+0.6758I+0.3242J	0.3254+0.3123I+0.3623J	0.3067+0.3060I+0.3873J	0.0915+0.9085I+0J	0.0916+0.9084I+0J	0.0846+0.9154I+0J
X6	0.3490+0.3029I+0.3481J	0.3740+0.6260I+0J	0.3016+0.2988I+0.3996J	0.3081+0.2993I+0.3926J	0.1731+0.8269I+0J	0.1731+0.8269I+0J	0.1731+0.8269I+0J
X7	0.3676+0.3105I+0.3219J	0.4812+0.5188I+0J	0.3093+0.2982I+0.3925J	0.2955+0.2952I+0.4093J	0.1517+0.8483I+0J	0.1518+0.8482I+0J	0.1443+0.8557I+0J
X8	0.4129+0.5871I+0J	0+0.6757I+0.3243J	0.3253+0.3123I+0.3624J	0.3067+0.3060I+0.3873J	0.0913+0.9087I+0J	0.0914+0.9086I+0J	0.0844+0.9156I+0J
X9	0.4636+0.5364I+0J	0.2818+0.2442I+0.4740J	0+0.5079I+0.4921J	0.4953+0.2195I+0.2852J	0.3760+0.2333I+0.3907J	0.4261+0.5739I+0J	0.3899+0.6101I+0J

Table S3 The connection entropy and total entropy of indicators of the actual water-use schemes from 2006 to 2016 and the optimal water resources allocation schemes for the three groundwater exploitation modes

Year	X1	X2	X3	X4	X5	X6	X7	X8	X9	The toal entropy
2006	0.14	0.18	0.16	0.20	0.10	0.16	0.17	0.10	0.19	1.40
2007	0.14	0.18	0.01	0.11	0.20	0.23	0.27	0.20	0.18	1.52
2008	0.15	0.18	0.21	0.02	0.07	0.23	0.30	0.07	0.11	1.34
2009	0.05	0.20	0.16	0.15	0.11	0.24	0.22	0.11	0.15	1.39
2010	0.02	0.20	0.17	0.21	0.15	0.29	0.29	0.15	0.16	1.64
2011	0.08	0.00	0.19	0.08	0.06	0.09	0.10	0.06	0.09	0.75
2012	0.10	0.01	0.01	0.07	0.08	0.09	0.10	0.08	0.01	0.55
2013	0.02	0.01	0.02	0.16	0.02	0.10	0.09	0.02	0.01	0.45
2014	0.10	0.04	0.02	0.21	0.15	0.03	0.02	0.14	0.1	0.81
2015	0.03	0.01	0.11	0.07	0.07	0.12	0.12	0.07	0.21	0.81
2016	0.07	0.01	0.01	0.1	0.08	0.11	0.12	0.08	0.05	0.63
Groundwater extraction mode 1	0.07	0.16	0.01	0.04	0.05	0.06	0.07	0.05	0.07	0.58
Groundwater extraction mode 2	0.07	0.01	0.02	0.04	0.05	0.06	0.07	0.05	0.02	0.39
Groundwater extraction mode 3	0.07	0.01	0.01	0.04	0.05	0.06	0.07	0.05	0.02	0.38
